# Assessment of Self-Medication Awareness During Pregnancy Among Saudi Women in Madinah: A Cross-Sectional Study

**DOI:** 10.7759/cureus.82078

**Published:** 2025-04-11

**Authors:** Abdulhakim M Alhazmi, Mohammed A Elmuttalut, Faris M Elmahdi, Joman M Mahrous, Seba M Alahmadi, Shahad E Abutowaimah

**Affiliations:** 1 Clinical Sciences, College of Medicine, Al-Rayan National Colleges, Madinah, SAU; 2 Clinical Pharmacy, College of Health Sciences, Al-Rayan National Colleges, Madinah, SAU; 3 Basic Sciences, College of Medicine, Al-Rayan National Colleges, Madinah, SAU; 4 College of Medicine, Al-Rayan National Colleges, Madinah, SAU

**Keywords:** awareness, over-the-counter drugs, pregnancy, saudi arabia, self-medication

## Abstract

Background

Self-medication during pregnancy constitutes a considerable public health issue, presenting potential hazards to both maternal and fetal health. Notwithstanding global awareness initiatives, there is a paucity of research on self-medication habits among Saudi women, especially in Madinah.

Objective

The study aims to assess awareness levels of self-medication during pregnancy and identify primary factors affecting knowledge among Saudi women in Madinah.

Methods

A cross-sectional study was performed at designated healthcare facilities in Madinah from October to December 2024. A validated questionnaire was conducted with 400 pregnant women via face-to-face interviews. We classified awareness scores as poor, moderate, or good and conducted statistical analysis using IBM SPSS Statistics for Windows, Version 26 (Released 2019; IBM Corp., Armonk, New York, United States).

Results

Among the 400 participants, 194 (48.5%) were aged between 31 and 40 years, and 182 (45.5%) held bachelor’s degrees. The most used over-the-counter (OTC) medications were analgesics/antipyretics (369, 92.4%) and folic acid (394, 98.5%). Only 50 (37.5%) demonstrated good awareness of self-medication risks. Higher education (p = 0.001), employment (p = 0.028), and higher income (p < 0.001) were significantly associated with increased awareness.

Conclusion

Although the majority of pregnant women had a moderate understanding of self-medication hazards, targeted educational initiatives are essential, especially for individuals with lower socioeconomic levels. Enhancing public health policies and awareness initiatives can mitigate the hazards associated with self-medication during pregnancy.

## Introduction

The World Health Organization (WHO) defines self-medication as "the use of drugs to treat self-diagnosed disorders or symptoms, or the intermittent or continued use of a prescribed drug for chronic or recurrent disease or symptoms" [1].

During pregnancy, women commonly experience nausea, vomiting, headaches, and constipation, often leading them to self-medicate with over-the-counter (OTC) drugs or herbal remedies [2]. However, self-medication without professional guidance poses significant risks to both maternal and fetal health, including congenital disabilities, preterm birth, and developmental complications [3].

As the U.S. Food and Drug Administration (FDA) categorizes medications into five pregnancy risk groups (A, B, C, D, and X), with only a few OTC drugs classified as completely safe (Categories A and B) [4-5]. Despite these classifications, studies have shown that 40% of pregnant women in Saudi Arabia use OTC medications without medical consultation, highlighting a critical gap in awareness [6]. 

A previous study revealed that self-medication without a prescription is a significant and growing global public health issue in treating billions of health conditions annually [7]. Across different regions, the prevalence of self-medication varies widely, ranging from 38.5% to 92%, indicating that a significant portion of the global population uses drugs without proper medical consultation, with around 80% of drugs purchased without a prescription in developing countries [7]. Furthermore, estimates suggest that 7-55% of pregnant women in various Middle Eastern areas use herbal medicine [6].

Self-medication is a common practice in the Kingdom of Saudi Arabia (KSA), which has the highest rate of OTC medication use compared to other countries in the Arabian Peninsula region. A high number of pregnant females are using OTC medications, but there is still a lack of awareness regarding the appropriate use of these medications, which could lead to several major complications for both the mother and the fetus [6-8].

While several international studies have explored self-medication awareness during pregnancy, Saudi Arabia, particularly Madinah, has conducted limited research on this topic. This study seeks to address this gap by assessing awareness levels, identifying predictors of self-medication knowledge, and providing recommendations for targeted health interventions.

## Materials and methods

Study design and setting

A cross-sectional study was conducted from October to December 2024 across antenatal care centers at selected healthcare facilities in Madinah, including primary healthcare centers (PHCs) and maternity hospitals under the Ministry of Health (MOH). The study aimed to assess the awareness and practices related to self-medication and vitamin use among pregnant women.

Study population and sampling technique

A total of 400 Saudi pregnant women participated in the study. A cluster sampling technique was employed to select participants from healthcare facilities providing antenatal care. The required sample size was calculated using Epi Info Software (version 7.2.5.0) with a 95% confidence level and a 5% margin of error.

Data collection

Data was collected through face-to-face interviews using a structured, validated questionnaire. The questionnaire was divided into three main sections: demographic information, medical history, and awareness assessments. The demographic section included questions on age, marital status, education level, employment status, and monthly income. The medical history section gathered information on the presence of chronic diseases and pregnancy-related history. The awareness section consisted of a 14-item assessment on self-medication and a four-item assessment on folic acid knowledge. Responses to the awareness questions were coded as correct (1) or incorrect (0).

Inclusion and exclusion criteria

The study included pregnant women attending antenatal clinics who were of Saudi nationality and agreed to participate by signing the informed consent form. Women who refused to participate or did not meet the inclusion criteria, such as non-Saudi nationals, were excluded from the study.

Questionnaire criteria

Awareness toward self-medication during pregnancy has been assessed using a 14-item anonymous questionnaire (9-10), with the correct answer for each questionnaire identified and coded with 1, while the incorrect answer has been coded with 0. The total awareness score has been calculated by adding all 14 items. Scores ranging from 0 to 14 points have been generated. The higher the score, the higher the awareness about self-medication use during pregnancy. By using 50% and 75% as cutoff points to determine the level of awareness, women were considered to have poor awareness if the score was less than 50%, 50% to 75% as moderate, and above 75% as a good awareness level (11).

Statistical analysis

The data were analyzed using the IBM SPSS Statistics for Windows, Version 26 (Released 2019; IBM Corp., Armonk, New York, United States). Descriptive statistics were presented using numbers and percentages (%) for all categorical variables, while mean and standard deviation were used to elaborate continuous variables. The differences in awareness scores among the sociodemographic characteristics of the women have been conducted using the Mann-Whitney Z-test and Kruskal-Wallis H-test. The normality test was performed using the Kolmogorov-Smirnov test. Based on the plot, the awareness score follows a non-normal distribution. Therefore, the non-parametric tests were applied. Values were considered significant with a p-value of less than 0.05.

Ethical considerations

Ethical approval has been obtained from the academic, training, and research affairs administration in the Madinah health cluster in the MOH IRB log (24-104). Informed consent was obtained from all participants; their information will be kept confidential with no identifiers. The confidentiality of the research data will be maintained throughout the research process, and it will be saved for three years on a laptop with a password.

## Results

This study included 400 Saudi women. Table 1 outlines the sociodemographic characteristics of the participants. The majority of the women, 194 (48.5%), were aged between 31 and 40 years. Nearly all participants, 394 (98.5%), were married, and 182 (45.5%) held a bachelor's degree. Most of the respondents, 306 (76.5%), identified as housewives. In terms of monthly income, 198 (49.5%) earned less than 5,000 SAR per month. The prevalence of chronic diseases among women was 62 (15.5%). Additionally, 90 (22.5%) were experiencing their first pregnancy, while 177 (44.3%) had three or more children. Only 11 (2.8%) reported having a child with special needs, and 170 (42.5%) had a history of miscarriage.

**Table 1:** Sociodemographic characteristics of the Saudi women (n = 400)

Study variables	N (%)
Age group	
15-20 years	02 (0.50%)
21-30 years	160 (40.0%)
31-40 years	194 (48.5%)
>40 years	44 (11.0%)
Marital status	
Married	394 (98.5%)
Divorced	04 (01.0%)
Widowed	02 (0.50%)
Educational level	
No degree	52 (13.0%)
High school graduate or diploma	155 (38.8%)
Bachelor's degree	182 (45.5%)
Postgraduate	11 (02.8%)
Occupation	
Student	19 (04.8%)
Housewife	306 (76.5%)
Healthcare provider	15 (03.8%)
Employee	60 (15.0%)
Monthly income (SAR)	
<5,000	198 (49.5%)
5,000-10,000	113 (28.2%)
>10,000	89 (22.3%)
Do you have any chronic disease? (hypertension, Diabetes, asthma or other)	
Yes	62 (15.5%)
No	338 (84.5%)
Is this your first pregnancy?	
Yes	90 (22.5%)
No	310 (77.5%)
How many children do you have?	
None	80 (20.0%)
One	63 (15.8%)
Two	80 (20.0%)
Three or more	177 (44.3%)
Have you had a child with special needs? Hereditary disease?	
Yes	11 (02.8%)
No	389 (97.3%)
Have you experienced a miscarriage?	
Yes	170 (42.5%)
No	230 (57.5%)

The assessment of awareness regarding self-medication during pregnancy revealed that 287 (71.8%) participants understood the meaning of OTC drugs, and 342 (85.5%) correctly believed that not all OTC drugs are safe during pregnancy. A majority of participants, 281 (70.3%), reported never using OTC medications without a prescription during pregnancy. Additionally, 350 (87.5%) recognized that the first trimester is the most critical period for fetal development and the most dangerous time to take medications. When it came to reading medication information, 267 (66.8%) stated they usually read the information leaflet provided with medications.

Regarding sources of information about OTC medications, including vitamins, 158 (39.5%) relied on physicians, 173 (43.3%) consulted pharmacists, and 58 (14.5%) used government organization websites. In contrast, 318 (79.5%) did not rely on relatives or friends, and 277 (69.3%) did not use social media or Google for such information. Furthermore, 336 (84.0%) participants did not believe that natural remedies are safe during pregnancy, and 298 (74.5%) expressed a need for more information about using OTC drugs, including vitamins and minerals, during pregnancy (Table 2).

**Table 2:** Assessment of awareness about using self-medication and folic acid during pregnancy (n = 400)

Awareness about self-medication	N (%)
Do you know what is meant by over-the-counter drugs? (yes)	287 (71.8%)
Do you think that all over-the-counter drugs are safe to be taken during pregnancy? (no])	342 (85.5%)
Have you ever used an over-the-counter medication without a prescription during pregnancy? (no)	281 (70.3%)
At which stage of pregnancy is taking medication most dangerous for the fetus? (first trimester)	350 (87.5%)
Do you usually read the information leaflet of the medications you use? (yes)	267 (66.8%)
What is your source for getting information on using over-the-counter medication, including vitamins?	
Relatives or friends (no)	318 (79.5%)
Physician (yes)	158 (39.5%)
Pharmacist (yes)	173 (43.3%)
Government organization websites (yes)	58 (14.5%)
Social media, Google (no)	277 (69.3%)
Do you think natural remedies are safe to be taken during pregnancy? (no)	336 (84.0%)
Do you feel you need more information about using (over-the-counter drugs), including vitamins and minerals, during pregnancy?‎ (yes)	298 (74.5%)
Did you use any type of vitamins or minerals during pregnancy without consulting your doctor? (no)	360 (90.0%)
Do you think that all vitamins and minerals are safe to be taken during pregnancy? (no)	327 (81.8%)
Total awareness score (mean ± SD)	9.58 ± 2.01
Level of awareness	
Poor	31 (07.7%)
Moderate	219 (54.8%)
Good	150 (37.5%)

The majority of participants, 360 (90.0%), reported not using any vitamins or minerals during pregnancy without consulting a doctor, and 327 (81.8%) correctly believed that not all vitamins and minerals are safe to take during pregnancy. The overall mean awareness score was 9.58 ± 2.01, with awareness levels categorized as poor (31, 7.8%), moderate (219, 54.8%), and good (150, 37.5%).

In terms of folic acid awareness, only 76 (19.0%) correctly identified folic acid as a vitamin, while 215 (53.8%) knew that folic acid helps prevent neural tube defects. Additionally, 175 (43.8%) were aware that folic acid should be taken three months before pregnancy, and 283 (70.8%) recognized that the first trimester is the most appropriate time to take folic acid.

Measuring the differences in awareness scores among the sociodemographic characteristics of the women found that higher awareness scores were associated with having higher education (Z = 3.317; p = 0.001), being employed/student (Z = 1.916; p = 0.028), increasing monthly income (Z = 3.489; p < 0.001), and no previous history of miscarriage (Z = 2.375; p = 0.018). No significant differences were observed between awareness scores in terms of age, associated chronic disease, first pregnancy, number of children, and having a child with special needs (p > 0.05) (Table 3).

**Table 3:** Differences in awareness score and the sociodemographic characteristics of the Saudi women (n = 400) ^§^ p-value has been calculated using the Mann-Whitney Z-test.^ ‡^ p-value has been calculated using the Kruskal-Wallis H-test. ^** ^Significant at p < 0.05 level

Factor	Awareness score (14) mean ± SD	Z-test	p-value ^§^
Age group			
≤30 years	9.48 ± 1.97	1.153	0.249
>30 years	9.65 ± 2.05		
Educational level			
Diploma or below	9.26 ± 2.11	3.317	0.001 **
Bachelor or higher	9.93 ± 1.85		
Occupation			
Employed/student	9.98 ± 1.69	1.916	0.028 **
Housewife	9.46 ± 2.09		
Monthly income (SAR)			
<5,000	9.18 ± 2.19	3.489	<0.001 **
≥5,000	9.98 ± 1.74		
Associated chronic disease			
Yes	9.27 ± 2.17	1.032	0.302
No	9.64 ± 1.98		
Is this your first pregnancy?			
Yes	9.70 ± 1.89	0.292	0.771
No	9.55 ± 2.05		
How many children do you have? ^‡^			
None	9.78 ± 1.86	1.764	0.184
1 - 2	9.74 ± 1.82		
≥3	9.36 ± 2.21		
Have you had a child with special needs? Hereditary disease?			
Yes	10.0 ± 2.05	0.576	0.565
No	9.57 ± 2.02		
Have you experienced a miscarriage?			
Yes	9.28 ± 2.15	2.375	0.018 **
No	9.80 ± 1.89		

**Figure 1:**
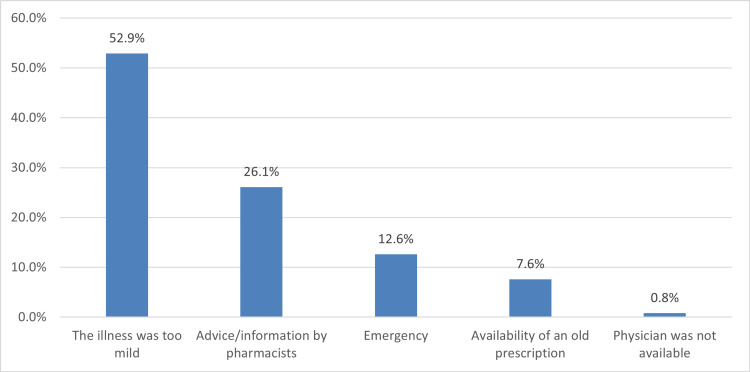
Reason for using OTC medication during pregnancy OTC: over the counter

Figure 1 depicts that the most common reason for using OTC medication during pregnancy was having a mild illness (211, 52.9%), followed by advice from the pharmacists (104, 26.1%), and emergencies (50, 12.6%).

**Figure 2:**
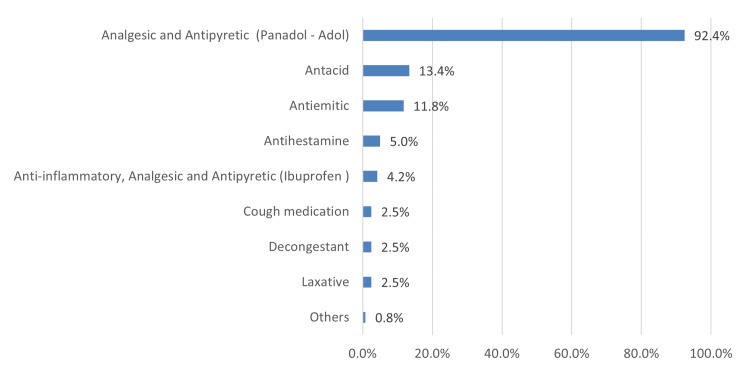
Type of medication being used without prescription

Figure 2 illustrates that analgesics and antipyretics were dominantly used during pregnancy (369, 92.4%), followed by antacids (54, 13.4%) and antiemetics (47, 11.8%).

**Figure 3:**
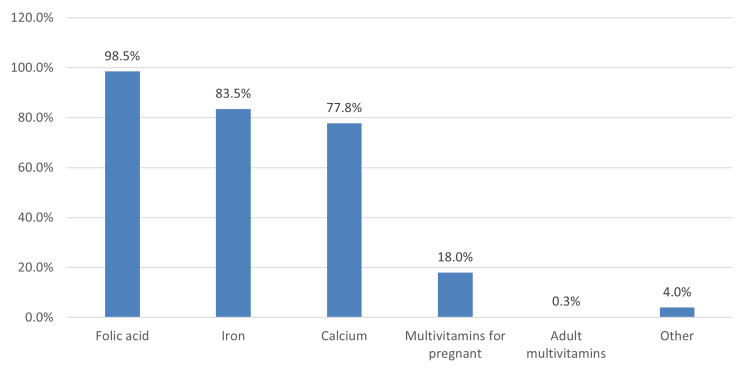
Use of vitamins and minerals during pregnancy

In Figure 3, multiple-response answers indicated that the most commonly used vitamins and minerals during pregnancy were folic acid (394, 98.5%), followed by iron (334, 83.5%) and calcium (311, 77.8%).

## Discussion

This study explores pregnant women's awareness of self-medication practices during pregnancy. Research of its kind could be an essential contribution to the publication. Taking medication during pregnancy without a prescription is a critical public issue, as it can lead to maternal and fetal adverse outcomes. Hence, awareness of pregnant women is critical to prevent adverse events associated with self-medication.

A significant factor in awareness

The findings of this study indicate that education, employment, and income levels significantly impact self-medication awareness among pregnant women, further suggesting that pregnant women with higher education, those who are currently employed or students, those with higher monthly income, and those without a previous history of miscarriage were associated with better awareness levels than other pregnant women. Highly educated women may have better awareness levels than those with lower education because they have better access to health information, greater exposure to healthcare systems, and more cautious attitudes toward medication use. Women who are employed or students may possess better awareness levels due to more exposure to workplace health policies, more social and professional networks, and financial stability. Further, women with higher economic status tended to have regular prenatal care, access to safer alternatives, and better insurance coverage than women with lower economic status, leading to improved awareness among them. Additionally, women without a previous history of miscarriage could demonstrate better awareness of self-medication during pregnancy due to the fact that they experienced fewer emotional disorders, fewer complications, and more positive experiences during pregnancy. A study by Atmadani et al. found a direct link between self-medication and knowledge about OTC. On the other hand, pregnant women who knew the risks of self-medication tended not to do it [5]. In contrast, Pereira et al. found that pregnant women with a high school or university education were more likely to use self-medication than pregnant women with less education [3]. Alani et al. found that age was significantly linked to the level of knowledge about self-medication during pregnancy [9-10]. In our study, age, having a chronic disease, being pregnant for the first time, having a lot of children, or having a child with special needs were not found to be significant predictors of awareness (p > 0.05), which was different from what other studies had found. Sample size determination, population diversity, methodology, and educational intervention influenced these variations.

Level of awareness

This study finds favorable awareness among pregnant women. Based on the given criteria, the overall mean awareness score was 9.58 out of 14 points. Stratifying the score, we noted that 92.3% were considered to have moderate to good awareness levels, and only about 8% were considered poor. Consistent with our findings, several studies documented a favorable understanding of pregnant women regarding self-medication [11-13]. This, however, was contradicted by the study of Alani et al., who reported that more than half of pregnant women had poor knowledge about medication practices during pregnancy [14]. These differences may vary according to region, sample population, study methodology, and healthcare access. Awareness of self-medication practices during pregnancy is necessary among women. This could lead to better maternal and fetal health outcomes. Hence, regular prenatal care visits are necessary to obtain appropriate advice from the healthcare provider regarding self-medication use.

Specific details of awareness

Most women were aware of the basic facts related to OTC medications. For instance, women demonstrated a high level of knowledge about the correct meaning of OTC drugs. Most of them held the belief that taking OTC drugs during pregnancy is not safe. Approximately 90% were correct that during the first trimester, taking medications was dangerous for the fetus, and about two-thirds usually read information leaflets enclosed in the medication. Despite their harmless nature, women do not consider natural remedies safe for use during pregnancy. Likewise, women were of the opinion that not all vitamins and minerals are safe to be taken during pregnancy and should not be taken without a doctor's consent. Finally, approximately 70% of pregnant women were not using OTC medication without a prescription. This is in agreement with the previous reports of Abduelkarem and Mustafa [9]. Most women agreed that "not all OTC drugs are safe to be taken during pregnancy," despite 40% of them reporting taking OTC medication during pregnancy [14]. In contrast, a more recent study by Nirmani et al. revealed misconceptions about OTC medications [13]. A total of 93% of the respondents had a wrong perception that medicine can be used at any stage of pregnancy, and more than one quarter had no idea that the medication taken during pregnancy could be harmful to the fetus. Additionally, about 87% of the women incorrectly believed that they could purchase antibiotics without a prescription [13]. Banzal et al. reported the excessive use of OTC medications during pregnancy. Approximately three-quarters of pregnant women were taking some kind of medication at the time of the survey, with more than half (51%) taking them regularly [15]. These differences could be due to regional settings, population characteristics, cultural influences, and methodological approaches. Improper practices of taking medication during pregnancy can have serious implications for both the mother and the developing baby. Hence, awareness campaigns are critical to educating women about the risk of self-medication during pregnancy.

Sources of OTC medication information

Our study also highlights that social media and nonmedical sources remain prevalent sources of medication advice, raising concerns about misinformation. While 43.3% of participants cited pharmacists as a source of information, only 39.5% relied on physicians. These findings underscore the need for stronger engagement between healthcare providers and pregnant women to ensure accurate information dissemination. Potential reasons for this effect were the lack of accessibility and time constraints, the lack of engagement among physicians, the delay in updates on government websites, and concerns about misinformation. This agrees with the study of Al-Ghamdi et al., suggesting that friends and family were the primary sources of information related to OTC medications [7]. This is contrary to the study of Kumari et al., which suggested that Indian pregnant women preferred physicians' advice before taking OTC medication [16], while Egyptian women preferred pharmacists as a source for self-medication practices [17]. Different focus, population diversity, and methodologies were some of the reasons for these variations. Seeking advice about self-medication during pregnancy through proper channels could ensure the safety of the mother and the baby. Women with pregnancy-related complications are at higher risk due to a lack of knowledge of the medications. Hence, physicians' advice is vital to avoid such pregnancy-related adverse events.

The purpose of using OTC medication during pregnancy

Pregnant women's most common reason for taking OTC medication during pregnancy was mild illness. The most common reasons for using OTC medication during pregnancy vary depending on the region. For example, Alyami et al. [10] indicated mild illness as the main reason for using OTC drugs. However, among Egyptian pregnant women consisting of 1050 cohorts, lack of access to medication in governmental healthcare facilities and the health provider's lack of concern to complaints were the primary reasons for using self-medication, while among Saudi women living in Al-Kharj, the challenge of reaching hospitals and the lack of efficiencies of health centers in providing necessary care are the prominent reasons for self-medication. Awareness of the potential risks and benefits of OTC medications may lead to better decisions about using them. Ultimately, the use of OTC drugs during pregnancy should always be guided by healthcare professionals to avoid any adverse effects on the pregnancy.

Study limitations

This study is limited by its hospital-based sampling, potentially excluding pregnant women who do not seek antenatal care. Additionally, reliance on self-reported data introduces the risk of recall bias.

## Conclusions

The study found that while many pregnant women exhibit moderate awareness of self-medication risks, significant knowledge gaps remain, particularly among those with lower education and income. Public health initiatives should focus on integrating self-medication education into routine antenatal visits and expanding pharmacist-led counseling programs. Future research should explore digital health tools as a means of improving awareness and accessibility to reliable medical guidance.
